# Preoperative Carbohydrate Loading on Outcomes after Cardiac Surgery: A Flawed Meta-Analysis. Comment on: “The Effect of Preoperative Carbohydrate Loading on Clinical and Biochemical Outcomes after Cardiac Surgery: A Systematic Review and Meta-Analysis of Randomized Trials”. *Nutrients* 2020, *12*, 3105

**DOI:** 10.3390/nu12123904

**Published:** 2020-12-21

**Authors:** Dileep N. Lobo, Girish P. Joshi

**Affiliations:** 1 Gastrointestinal Surgery, Nottingham Digestive Diseases Centre, National Institute for Health Research (NIHR) Nottingham Biomedical Research Centre, Nottingham University Hospitals NHS Trust and University of Nottingham, Queen’s Medical Centre, Nottingham NG7 2UH, UK; 2 MRC Versus Arthritis Centre for Musculoskeletal Ageing Research, School of Life Sciences, University of Nottingham, Queen’s Medical Centre, Nottingham NG7 2UH, UK; 3 Department of Anesthesiology and Pain Management, University of Texas Southwestern Medical Center, Dallas, TX 75390-9068, USA; girish.joshi@utsouthwestern.edu

We read, with interest, the publication in *Nutrients* on the effects of preoperative carbohydrate loading on outcomes after cardiac surgery [1] and wish to highlight several inconsistencies and shortcomings that make the conclusions unreliable.

The protocol was not registered with an appropriate registry and, hence, the study is non-compliant with the PRISMA statement [2]. Lack of registration introduces bias, as it is possible for the end points to be changed depending on the results obtained. In addition, there should be a single stated primary end-point, with the other predetermined end-points being secondary. The authors [1] have chosen a host of primary outcome measures, and it is difficult to envisage how preoperative carbohydrate loading could influence some of these. For example, the decrease in aortic cross clamping time is more likely to be a technical issue rather than a beneficial metabolic effect of carbohydrate loading and, in turn, could have influenced other outcomes such as durations of cardiopulmonary bypass, mechanical ventilation and intensive care unit (ICU) stay.

There is also significant heterogeneity in the included studies. Cardiac surgery was performed off-pump in two studies [3,4], and this is known to induce less stress than cardiopulmonary bypass. The volume of carbohydrate loading ranged from 200 to 1200 mL, and the timing of administration varied considerably. The preparation of carbohydrate used was not uniform as two studies used maltodextrin in combination with omega-3 fatty acids [5,6]. The comparator in the control group varied: administration of water versus fasting. A recent meta-analysis has suggested that carbohydrate loading provides no benefits over adequate preoperative hydration [7].

No information has been provided on perioperative care or use of Enhanced Recovery After Surgery (ERAS) pathways [8] in the studies included. There could have been a wide variability in perioperative care including postoperative oral intake, thus introducing further bias [9,10].

Two of the trials with the same trial registration number (NCT03017001) [5,6] had overlapping patients and, therefore, inclusion of both is methodologically incorrect and weakens the meta-analysis. Ideally, only the larger trial should have been included [6].

In the conclusions, the authors mistakenly state that preoperative carbohydrate loading in patients undergoing cardiac surgery demonstrated a 50% reduction in length of ICU stay and a 28% decrease in aortic clamping time. However, what the results actually show is that the standardized mean reductions in ICU stay and aortic clamping time were 54 h and 28 min, respectively. The authors also state that patients who received carbohydrate loading required 35% less insulin postoperatively than controls. This was in a subgroup of only 85 patients, and the actual standardized mean difference in insulin requirements was 0.349 IU. While this difference may have been statistically significant, it begs the question of whether it was clinically relevant.
